# Dairy cows value an open area for lying down

**DOI:** 10.1371/journal.pone.0268238

**Published:** 2022-05-27

**Authors:** Laura Shewbridge Carter, Marie J. Haskell, David Ball, Jenny Gibbons, W. Edwin Harris, S. Mark Rutter

**Affiliations:** 1 Department of Veterinary Health and Animal Sciences, Harper Adams University, Edgmond, Newport, Shropshire, United Kingdom; 2 Animal and Veterinary Sciences, Scotland’s Rural College, Edinburgh, United Kingdom; 3 Agriculture and Horticulture Development Board, Stoneleigh, Kenilworth, United Kingdom; 4 Department of Agriculture and Environment, Harper Adams University, Edgmond, Newport, Shropshire, United Kingdom; University of Illinois, UNITED STATES

## Abstract

As dairy cows are being housed for longer periods, with all-year-round housing growing in popularity, it is important to ensure housed environments are meeting the needs of cows. Dairy cows are motivated to access open lying areas, although previous motivation studies on this topic have confounded surface type and location (i.e. pasture outdoors vs cubicles indoors). This study measured cow motivation for lying down on an indoor open mattress (MAT; 9 m x 5 m) compared to indoor mattress-bedded cubicles, thus removing the confounding factor of surface type and location. This was repeated for an identically sized indoor deep-bedded straw yard (ST), to investigate whether surface type affected motivation for an open lying area. Thirty Holstein-Friesian dairy cows were housed in groups of 5 (n = 5 x 6) in an indoor robotic milking unit with access to six mattress-bedded cubicles. To assess motivation, cows were required to walk increasing distances via a one-way indoor raceway to access the open lying areas: Short (34.5 m), followed by Medium (80.5 m) and Long (126.5 m). Cows could choose to walk the raceway, leading to the MAT or ST, to lie down or they could lie down on the cubicles for ‘free’. Overall, cows lay down for longer on the open lying areas at each distance compared to the cubicles, with cows lying down slightly longer on ST than MAT, although lying times on the open lying areas did decrease at the Long distance. However, cows were still lying for >60% of their lying time on the open lying areas at the Long distance. This study demonstrates that cows had a high motivation for an open lying area, the provision of which could better cater for the behavioural needs of housed dairy cows and improve housed dairy cow welfare.

## Introduction

The majority of dairy cows are housed at some point throughout their lives (99% of British cows are housed at some point throughout the year [1] and many farmers are moving to year-round housing [2,3]).When housed, dairy cows are most commonly housed in cubicles, known as freestalls in North America, a housing system which originated from tie-stall designs [4]. Various studies have shown that lying down is an important behaviour to cows (Rebound response [5,6]; Trade-off: [7,8]; Operant conditioning [9,10]; Consumer demand [11]). Therefore, it has become increasingly important to establish whether cubicles are the best system to meet the behavioural and welfare needs of cows.

Motivation tests require animals to work to gain access to a valued resource in order to quantify the value the animal places on that resource. Tests of motivation are commonly preceded by a preference test [12]. Preference tests allow an animal to choose between differing variations of one resource or between different resources, with the assumption that the animal chooses in a way that best provides for their own welfare [12]. A great deal of research has been carried out on cow lying preference, investigating a range of cubicle modifications (stall width and length [13]; neck rail placement [14]; surface type [15,16]). Although cows appear more focused on the lying surface of a cubicle, preferring softer lying surfaces, a trade-off preference study found that when choosing where to lie down, an open lying space is more important to cows than their preferred lying surface [17]. Cow preference for cubicles has been compared against open lying areas, such as pasture [18,19] and pack areas, both indoors [20] and outdoors [21], with the results supporting the proposition that cows have a preference for an open lying area.

However, preference tests do not necessarily differentiate between the preference for one resource (suggesting preference is based on perceived benefits) versus the avoidance of another (suggesting preference is based on choosing the lesser of two evils) [12,22], i.e. the option chosen may represent avoidance of the other option(s), with true preference being for an option that is not presented in the test. Therefore, the motivation for a preference choice must be further investigated in order to quantify preference and establish whether the provision of the chosen resource actually does lead to improved welfare [23], or whether its absence leads to animal suffering [24], with the assumption that an animal with a strong motivation to perform a behaviour or to access a resource would experience suffering if it is prevented from performing that behaviour or accessing the resource [25]. For dairy cows, weighted gates [11,26] and distance walked [27–29] to gain access to a resource has been used to measure motivation, with increasing weight or distance, respectively, being the cost to cows for access. Studies have shown that cows are motivated to gain access to pasture, walking long distances [28,29] and pushing weighted gates for access [26]. This motivation has been shown to be higher at night [26,28,29]. As the amount of pasture available for grazing has been shown to have no effect on this motivation [29], it has been suggested that motivation for pasture access is primarily being driven by lying behaviour, possibly due to the open space pasture offers for lying. However, cow motivation for pasture, as an open lying area, has always been tested against indoor cubicles, with the results confounded by surface type and/or location of the lying areas provided (i.e., pasture open lying area outdoors vs mattress cubicle lying area indoors). Therefore, it remains unclear whether this motivation to access an open lying area, in the form of pasture, is due to it being outdoors or because of the surface it provides for lying down compared to what is offered indoors.

The aim of the current study was to measure cow motivation for lying down on an indoor open mattress lying area [MAT] when cows had free access to indoor mattress-bedded cubicles, thus removing the confounding factors of surface type and location. This was repeated for a deep-bedded straw yard (ST) of an identical size to the open mattress, to further investigate the value cows place on lying surface type and the relationship between this and the value cows place on lying space. Walking distance was used as a measure of cow motivation to access these open lying areas via an indoor raceway, set at three different increasing distances (Short, 34.5 m; Medium, 80.5 m; Long 126.5 m). Given that previous studies appear to show that cows are motivated to access open lying areas, and particularly at night, we predicted that (1) cows would spend more time lying in the open lying areas compared to the cubicles at all walking distances, (2) that total lying times on the open lying areas would decrease with walking distance imposed and (3) that lying times at night on the open lying areas would not be effected by walking distance. Finally, we predicted that (4) surface type would also influence lying times on the open lying surfaces, with cows being more motivated to access the deep-bedded straw than the mattress.

## Materials and methods

Ethical approval for this study was given by the Harper Adams University Research Ethics Committee (0488-201905-PGMPHD).

### Animals and management

The study was carried out at the Agri-EPI Midlands Dairy Research Centre at Harper Adams University, Shropshire, United Kingdom, in an open-sided barn. Thirty pregnant Holstein-Friesian dairy cows were selected for the study (see S1 Table for cow details). Cows were randomly selected based on a confirmed pregnancy status assessed by the farm veterinarian, had a body condition score (BCS) between 2.75 and 3.5, as described by the Penn State method [30], and a lameness score (LS) no greater than 2 (2 = imperfect locomotion but ability to move freely not diminished [31]). BCS and LS were assessed by the same person (LSC) while cows walked across a concrete floor before cows came on trial. The cows were allocated to 1 of 6 experimental periods according to their stage of lactation, with groups being balanced for lactation number as much as possible (n = 5 x 6). Trials were carried out from August 31^st^ 2019 to July 21^st^ 2020 (see S2 Table for details). Before the study, the cows in this herd had been housed indoors in an open-sided cubicle barn on sawdust bedded rubber mats with plastic, flexible cubicle dividers. All cows had previous experience of a straw yard during the pre-calving period and metal cubicles as heifers. Cows had free access to one milking robot (VMS V200, De Laval).

### Trial area housing

**Fig 1:**
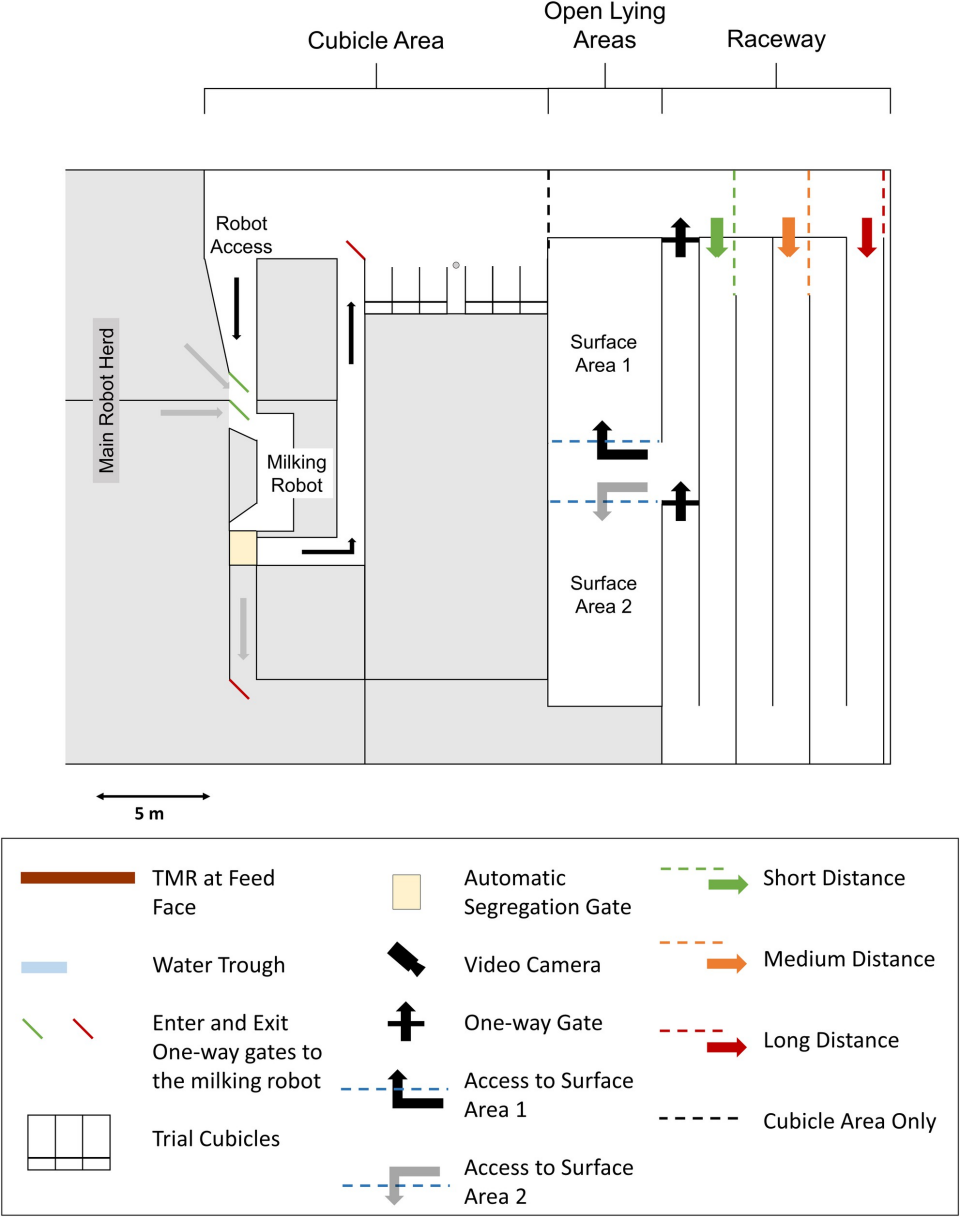
Plan of the trial area. The area is split into two main components; the ‘Cubicle Area’ and ‘Experimental Area’. Within the ‘Cubicle Area’ cows had access to six cubicles, feed face, a water trough and the milking robot. The ‘Experimental Area’ contained an indoor raceway which allowed access to one of two open lying areas at a time, ‘Surface Area 1’ and ‘Surface Area 2’. The raceway could be adjusted to three different distances, via the three difference entrances indicated, to a Short (34.5 m), Medium (80.5 m), and Long (126.5 m) distance.

The trial area was located in an area of 453-m^2^ at the south-east end of the open sided barn in which the cows were previously housed. The area used was a designated trial area within the barn and was not regularly used to house cows. On occasion, dry cows were previously housed in an overflow straw yard in this area, configured differently to the layout of the trial area of this study. The configuration of the trial area for this study was designed specifically for the study and was not familiar to the cows. Trial cows were separated from the main herd, of approximately 50 cows, into the trial area using cow hurdles. The trial area was split into two main areas (Fig 1). The ‘Cubicle Area’ included six sawdust-bedded cubicles with a mattress, purpose built for the study (2.7 x 1.2 m; Super Comfort Cubicles, Intershape Ltd., Daventry, England, UK; Pasture Mat; Wilson Agri, Coleraine, Northern Ireland, UK), access to the milking robot, and a feed-face and water-trough providing *ad libitum* Partial Mixed Ration (PMR) and clean water, respectively. PMR was provided daily at approximately 0730 h, with feed refusals being removed every day before fresh feed was provided. Cows were fed concentrates, based on milk yield, during milking in the milking robot.

The ‘Experimental Area’ (Fig 1) comprised of an indoor raceway (1.5 m wide) on a concrete floor, which could be adjusted to three different distances (Short; 34.5 m, Medium; 80.5 m, Long; 126.5 m), and incorporated a number of 180 degree turns (Short: 1 turn; Medium: 3 turns; Long: 5 turns). Each turning area was 2.5 m x 3m and had a rubber mat floor (EASYFIX MG Max 4, Agri & Industrial Rubber Ltd, Galway, Ireland). The raceway led to two open lying areas (9.0 x 5.0m) of different surface types (deep-bedded straw [ST] and sawdust bedded mattress [MAT] (Pasture Mat; Wilson Agri, Coleraine, Northern Ireland, UK)), separated by a central access point. Cows only had access to one of the open lying areas at a time. The raceway had one-way gates to ensure that once a cow walked the length of the raceway and entered the surface area, via a first one-way gate, it was not possible to return to the ‘Cubicle Area’ via the raceway, but could only return to the ‘Cubicle Area’ via a short race (9 m) through a second one-way gate, thus setting up a one-way system in and out of the surface areas.

### Experimental design

**Fig 2:**
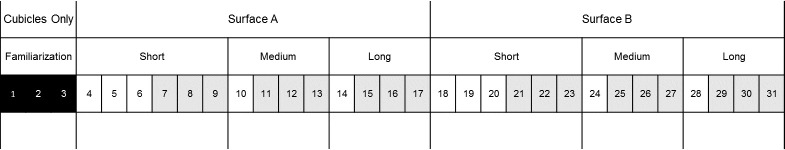
Representation for the 31-d study period, which was repeated 6 times using 5 cows per experimental period. Unshaded days represent training days and days shaded in gray represent choice periods. Days shaded in black represent the familiarization period, when cows did not have access to either open lying area. Short, Medium and Long describe the raceway distance in order to access the open lying areas, with Surface A being the first surface and Surface B being the second surface.

Throughout the study cows had continuous access to cubicles, with each experimental period lasting a total of 31-d (Fig 2): 3-d of familiarization (cubicle access only), followed by 14-d of access to the first open lying area (Surface A) and then 14-d of access to the second open lying area (Surface B), with cows only ever having access to one open lying area at a time.

While on trial, cows continued to have free access to the milking robot, were fed concentrates during milking, and were segregated into the trial area after milking via an automatic segregation gate (Fig 1). At approximately 0800 h each day, the trial area was cleaned out. When cows had access to the open lying areas, they were moved and kept in the cubicle area during cleaning.

Four video cameras (Swann, Milton Keynes, UK), were set up to continuously record cow behavior within the trial area (see Fig 1). Each experimental “day” began when the cows had access to the open lying areas after cleaning and ended when cleaning began the following day (approximately 1030–0800 h). Behaviour was only recorded during choice periods, as per Fig 2.

Each experimental period began with a 3-d familiarization period. During this time, cows only had access to the ‘Cubicle Area’ so that they could familiarize themselves with their new grouping, robot access and the cubicles. For the duration of the study, cows had ‘free’ access to the cubicles at all times and did not have to work to gain access to them.

During training periods, each cow was encouraged to walk the raceway distance, with a researcher walking quietly behind them, to gain access to the open lying space they had access to at the time. Following the familiarization period, the first training period, which always occurred at the Short distance, was 3-d long, to allow for one-way gate training (see S1 File for a detailed training protocol) as well as to allow cows to become familiar with the raceway length which had to be walked in order to access the first open lying area, Surface A. This training period was followed by a 3-d choice period, whereby cows had the choice to use the raceway to gain access to the open lying area available, paying the price of walking the Short distance (34.5 m), or could access the cubicles for ‘free’. The raceway length was then changed to the Medium distance (80.5 m; Fig 1), and cows had 1-d training, as per Fig 1. This was followed by another 3-d choice period. Finally, the raceway length was changed to the Long distance (126.5 m; Fig 1), and cows had a 1-d training period followed by a 3-d choice period. Then the lying surface choice access was changed from Surface A to Surface B and the above series of training and choice periods repeated.

For experimental periods 1–3, the open lying area marked ‘Surface Area 1’ as per Fig 1 was a mattress bedded with sawdust (MAT) and the open lying area marked ‘Surface Area 2’ was deep-bedded straw (ST). These surface locations were reversed for experimental periods 4–6; ‘Surface Area 1’ was ST and ‘Surface Area 2’ was MAT. For each experimental period, the order in which cows had access to surface type alternated; Group 1 had ST followed by MAT, Group 2 had MAT followed by ST, and so on.

### Performance and lameness

Throughout the study, milk yield was recorded automatically at each milking for individual cows via the robotic milking system and was used to calculate average yield per cow for the duration of their time on the study. Milking permission, in terms of milking frequency and latency between milkings, was managed by the herd manager depending on the stage of lactation for individual cows. The milking status of trial cows was monitored regularly by the herd manager and LSC, and any trial cows that had not successfully taken themselves to be milked for >12 h were encouraged to the robot for milking.

LS was evaluated after the trial area was cleaned each morning, at approximately 1000 h, on the first day of training for Surface A and Surface B (days 4 and 18 as per Fig 1) and on the first and last days of each choice period (the first and last days in the grey shaded blocks as per Fig 1).

### Data collection

For the ‘Cubicle Area’ during the choice periods, video footage was recorded from a camera located above the ‘Cubicle Area’ (see Fig 1) and was used to obtain cubicle lying bout start and end time for each cow. From this, total time spent lying and the number of lying bouts in the cubicles was calculated. For the ‘Experimental Area’ during the choice periods, video footage was analysed from cameras located above each surface area and above the raceway to obtain the enter and exit times to the open lying areas and the lying bout start and end times on the open lying areas. From this, times spent lying down and non-lying (standing and performing other behaviours while standing) in the open lying areas was calculated for each choice period for each cow. Additionally, frequency of raceway completions per choice period for each cow was recorded from this video data.

To account for changing daylight photoperiod during the trial, the time of sunrise and sunset was obtained from Time and Date [32], using geographical coordinates for the site of the trial area (52°46’52.8” N, 2°25’52.3” W). Time spent lying down during the day was defined as being between sunrise and sunset and time spent lying down during the night was defined as being between sunset and sunrise.

### Statistical analysis

Dependent variables were time spent lying, and not lying down on the open lying areas, and the time spent lying down in the cubicles versus open areas. The number of lying bouts was measured as the total lying bout frequency for each choice period. Lying bout duration was calculated as the average lying bout duration for each choice period. Lying bout frequency and average lying bout duration were calculated separately for open lying areas and the cubicles. Frequency of raceway completion on the open lying areas was measured as total raceway completion frequency for each choice period. Explanatory variables included chosen lying location (open lying, or cubicle), raceway distance (Short, Medium, or Long), and surface type for open areas (mat, or straw).

Linear mixed effects models were used to analyse the (fixed) effects of chosen lying location, raceway distance, and surface type. Assumptions of Gaussian residual distribution and homoscedasticity were examined and met for our analyses. Because treatments were applied at the group level, to meet the assumption of independence of observations (n groups = 6; n cows = 30; n cows per group = 5), group was treated as a random effect to identify it as the level of independent observation. All data were analysed using R version 4.1.0 [33] using the “lme4” package [34]. Main effects were evaluated using standard methods [35]. Degrees of freedom were estimated using Satterthwaite’s method [36] Comparisons of pairwise *post hoc* differences were made using the Sidak correction [37].

## Results

### Open lying areas versus cubicles

**Fig 3:**
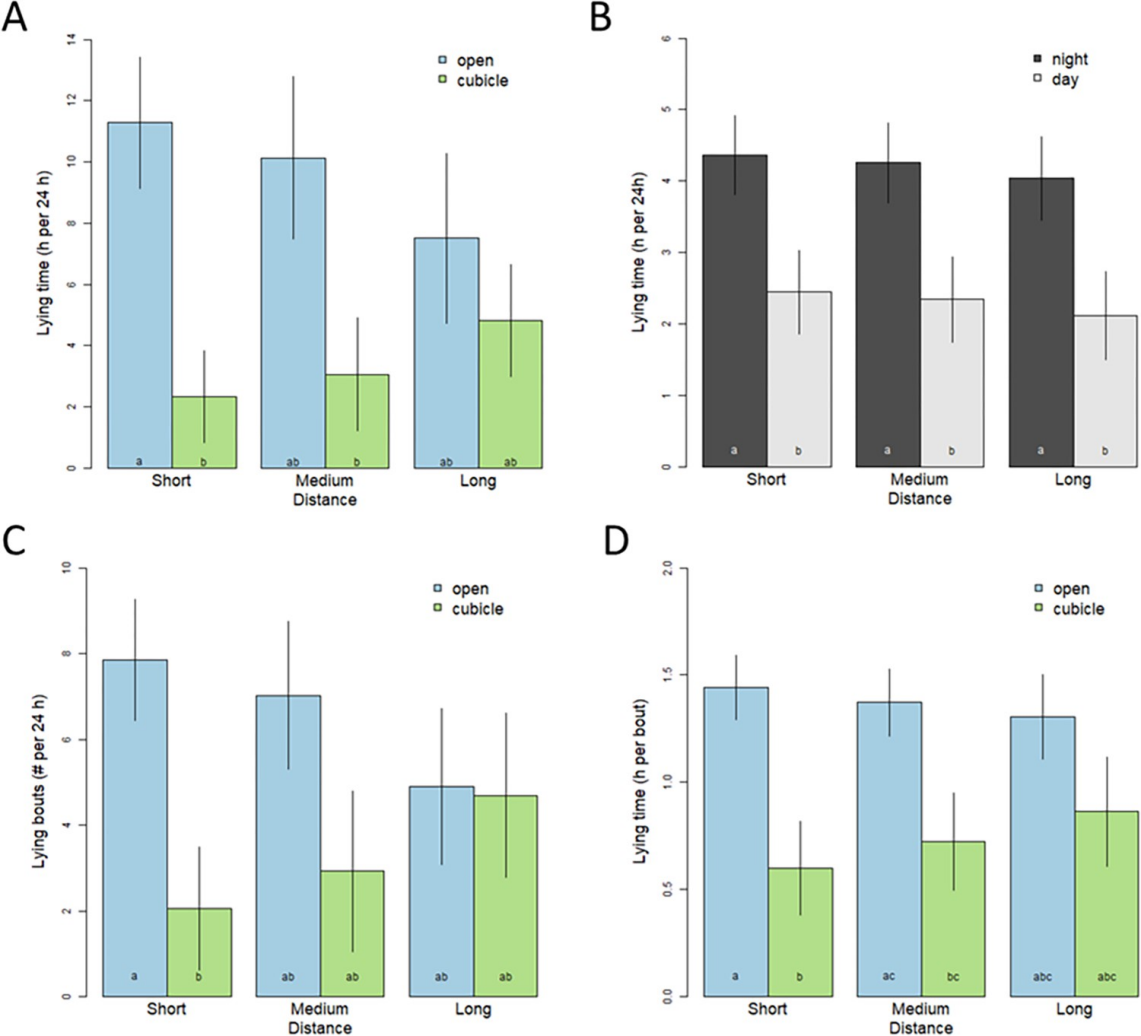
Lying as a function of distance choice treatment. Bar height represents the category mean and error bars represent the category 95% confidence interval (based on cow groups). Letters represent mean differences based on post hoc pairwise tests (Sidak corrected alpha = 0.05). A. Lying time per 24 h in open lying areas versus cubicles. B. Lying time per 24 h at night versus daytime. C. Frequency of lying bouts per 24 h in open lying areas versus cubicles. D. Average duration of lying bout per 24 h in open lying areas versus cubicles.

Overall lying time, lying bout frequency and lying bout duration results are shown in Fig 3 and were analysed in order to evaluate the effect of distance with respect to choices made while cows had access to the open lying areas and cubicles. An overall effect of location on lying time per 24 h was found, being higher in open lying areas than in cubicles (F_1,5.0_ = 11.53, P = 0.019; Fig 3A). Cows also spent more time lying at night than during the day (F_1,5.0_ = 19.25, P = 0.0071; Fig 3B). No significant average effect of distance on total lying time was found (F_2,46.1_ = 1.06, P = 0.35), but a significant interaction was detected between distance and location (F_2,5.7_ = 6.72, P = 0.032). As distance to access the open lying areas increased, lying time on the open lying areas decreased and increased on the cubicles. No overall effect of location on the frequency of lying bouts per 24 h was found (F_1,5.0_ = 5.47, P = 0.067; Fig 3C). No significant average effect of distance was found on the frequency of lying bouts (F_2,27.4_ = 0.08, P = 0.92), but there was a significant interaction between distance and location (F_2,6.4_ = 8.20, P = 0.018). A strong effect of location was also found on lying bout duration, with cows exhibiting longer lying bouts on the open lying areas than in the cubicles (F_1,5.0_ = 23.63, P < 0.005; Fig 3D). Here also, there was no significant average effect of distance found on the duration of lying bouts (F_2,12.4_ = 0.3, P = 0.77), nor was there a significant interaction between distance and location (F_2,7.6_ = 42.8, P = 0.12).

### MAT versus ST

**Fig 4:**
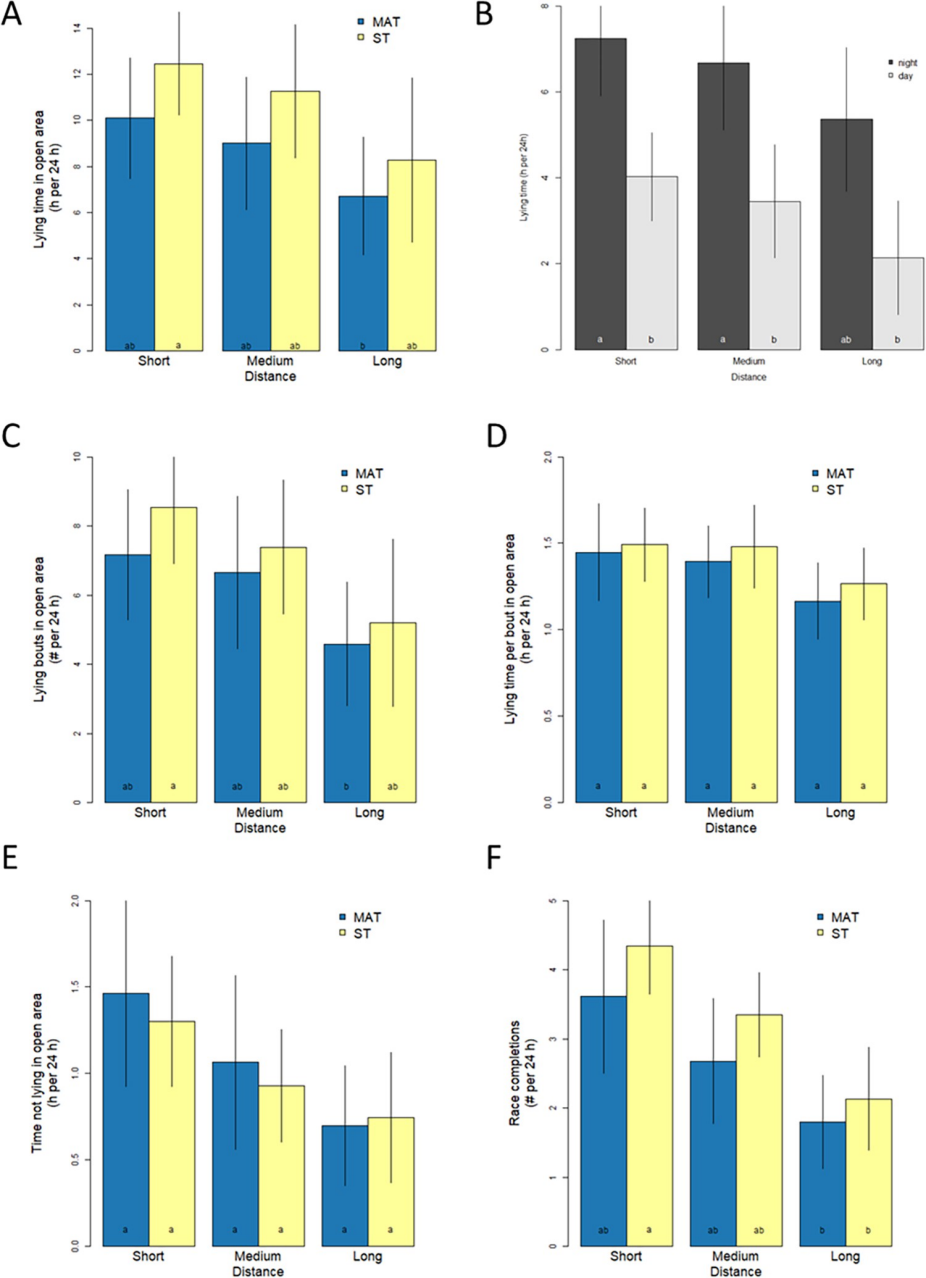
Lying, not lying and race completions as a function of distance choice treatment in open lying areas. Bar height represents the category mean and error bars represent the category 95% confidence interval (based on cow groups). Letters represent mean differences based on post hoc pairwise tests (Sidak corrected alpha = 0.05). Lying time is shown separately for when MAT or ST was available. A. Lying time per 24 h in open lying areas with MAT versus ST. B. Lying time per 24 h in open lying areas as night versus daytime. C. Frequency of lying bouts per 24 h with MAT versus ST. D. Average duration of lying bouts per 24 h in open lying areas with MAT versus ST. E. Time not lying per 24 h in open lying areas with MAT versus ST. F. Raceway completions per 24 h with MAT versus ST.

Lying time and other behaviors were also analysed with respect to choices made while in the open lying areas in order to evaluate the effect of surface type and distance (Fig 4). Distance was found to have a significant overall effect on lying time per 24 h, with lying time decreasing with increasing distance (F_2,6.7_ = 6.3, P = 0.029; Fig 4A). Cows spent more time lying at night than during the day (F_1,5.0_ = 27.59, P = 0.003; Fig 4B). Surface also had a strong effect on lying time, with cows choosing to lie longer on ST compared to MAT (F_1,5.7_ = 6.61, P = 0.044), and no significant interaction was found between distance and surface (F_2,15.5_, P = 0.82). Distance strongly influenced the frequency of lying bouts in the open lying areas, with the number of lying bouts decreasing with distance (F_2,11.0_ = 10.70, P 0.003; Fig 4C). There was no significant difference between the open lying areas in the frequency of lying bouts on ST compared to MAT (F_1,5.3_ = 1.60, P = 0.26), and no significant interaction between distance and surface (F_2,26.9_ = 0.25, P = 0.78). The mean duration of a lying bout was not higher for ST versus MAT (F_1,11.6_ = 4.16, P = 0.065; Fig 4D), nor was there a significant influence of distance (F_2,12.5_ = 0.65, P = 0.54) or a significant interaction between distance and surface (F_2,53.3_ = 0.37, P = 0.69). We found that the time spent not lying was also influenced by distance, with less time spent not lying as distance increased (F_2,11.1_ = 11.1, P = 0.012; Fig 4E). However, we did not detect a significant overall association between time spent not lying and surface type (F_1,8.8_ = 0.36, P = 0.4457) nor an interaction between distance and surface (F_2,6.7_ = 0.22, P = 0.81). Finally, the frequency of raceway completions was analysed and a strong decrease in the number of completions as distance increased was found (F_2,6.9_ = 19.6, P = 0.0014; Fig 4F), but we found no significant difference in the number of completions for MAT compared to ST (F_1,5.0_ = 1.42, P = 0.29). No interaction effect was found between distance and surface for the frequency of raceway completions (F_2,10.5_ = 10.5, P = 0.81).

## Discussion

The main objective of the study was to establish the extent of dairy cow motivation for lying on an open lying area when the cows had free access to cubicles, both of which were indoors and of the same surface type, (MAT; mattress bedded with sawdust), so as to remove these as confounding factors. This was repeated for a different surface type (ST; deep-bedded straw) to investigate the influence surface type has on motivation for an open lying area. Overall, time spent lying on the open lying areas declined as distance to access the open lying areas increased to the Long distance. However, cows still chose to lie down for longer on the open lying areas at this distance (>60% of lying time), compared to the free access cubicles, showing they were motivated to access the open lying areas rather than lying in cubicles. With a longer raceway we have observed shorter lying times for the open lying surfaces, but a ceiling effect (when the cost is too much and never paid) was not reached in the current study.

Surface type did influence motivation, with cows expressing a higher motivation and lying down longer on the open lying area when it was a deep-bedded straw yard (ST) compared to MAT. This could be due to a lack of previous experience the cows had with an open mattress lying area, whereas they did have previous experience of open straw yards during dry periods. An animal’s previous experience can affect the results of preference and motivation tests, altering an animal’s attraction or avoidance of either a familiar or unfamiliar resource [12,22]. Previous experience of cubicle surface type has shown to alter cow preference when choosing which surface to lie down on [15,16]. Additionally, both previous studies incorporated a resource exposure period within the study, 3d and 2d long, respectively, and found a change in preference results after this exposure, demonstrating the importance of previous experience. Similarly, cows had a 3d familiarisation period within the cubicle area at the start of each experimental period, along with a 3d training period on each open lying area at the Short raceway distance before behavioural measurements began. This allowed the cows to firstly become familiar with the cubicles within the trial area and then with the open lying areas, with regards to surface type, location, and raceway length. Therefore, the difference in lying times between ST and MAT may indicate a true preference for ST over MAT, although this difference may have been reduced if the cows had experienced a dry period on an open mattress area as well as in a straw yard.

Despite cows lying down longer on the ST compared to the MAT, there was no interaction between surface type and distance to access the open lying areas, suggesting that surface type had a limited effect on motivation and that access to an open lying area was the main driving factor for motivation in the current study. This is in agreement with a previous study whereby cows were found to trade lying down on their preferred lying surface with a cubicle for lying on an open lying space with a less preferred lying surface, demonstrating the increased value cows place on an open lying area than the lying surface [17]. An additional indicator of motivation is the number of times the cows in the current study completed the raceway to gain access to the open lying areas. In a 2018 study, a pneumatic push gate was used to measure cow motivation to lie down in a deep-bedded area when cows were deprived and not deprived of lying down [11]. They found that as the force required by the cows to push the gate open increased, the cows deprived of lying used the gate more frequently compared to non-deprived cows, demonstrating their motivation for lying. Furthermore, successful passes through the pneumatic gate decreased as it became more difficult to open. Similarly in the current study, as the distance to access the open lying areas increased, the number of successful raceway completions to access them decreased. However, there was no difference between the amount of raceway completions between the ST and MAT open lying areas, further supporting the idea that in the current study surface type had a more limited effect on cow motivation and access to an open lying area, regardless of surface type, was the main factor of cow motivation.

Unlike the relatively high motivation for an open lying area found in the current study, a previous study found that cows showed a relatively small and varied preference to lie down on a mattress open lying area (created by removing all cubicle hardware except for the brisket board) compared to cubicles of the same surface [20]. Both the previous study and the MAT treatment in the current study controlled for surface type, however cows still showed a high level of motivation for MAT in the current study compared to the low preference found in this previous study. The difference in results between the previous study and the relatively high motivation found in the current study may be due to a disparity in stocking densities between the two studies, in both the open lying areas and the cubicles. The current study allocated 9 m^2^ per cow on the open lying surfaces and had an 83% cubicle stocking density, compared to 3.1 m^2^ per cow on the open mattress area and 67% cubicle stocking density in the other study [20]. Cows have been shown to lie down for longer at lower cubicle stocking densities [38,39] and value space for lying down [17]. The lower stocking density of the open lying area in the current study may have made the open lying area more attractive for lying down, and subsequently the higher stocking density on the cubicles less attractive, compared to the previous study [20]. However, dry [40] and lactating [41] cows housed in straw yards with low (12m^2^ and 9m^2^ lying area per cow, respectively) and high stocking densities (6m^2^ and 4.5m^2^ lying area per cow, respectively) were found to have no difference in lying times. Therefore, it is possible that only the difference in cubicle stocking densities between these two studies accounts for the low preference in the previous study and high motivation in the current study for an open mattress lying area. These previous studies investigating the effects of stocking densities in straw yards on cow behaviour are relatively short term studies (40: 21d per cow; 41: 28d per experimental period) and further research is needed to investigate the long term effects on behaviour. Alternatively, the difference in results between that study and the relatively high motivation found in the current study may be due to the physical differences between the open lying areas. The current study offered cows a flat open lying area without obstruction, whereas the previous study offered cows a sloped lying area, due to the area’s previous function as cubicles, and the lying area was obstructed with the brisket board, limiting where the cows could lie down [20].

The total time cows spent in the open lying areas and lying on the open lying surfaces in the current study are similar to motivational studies for access to other open lying areas in the form of pasture access that also used walking distance as an indicator for motivation [28,29]. A 2014 study reported that, at a distance similar to the Short distance in the current study, cows spent longer at pasture compared to the open lying areas in the current study [29]. This difference may be due to the dual function of pasture as a lying and grazing area, resulting with cows spending more time at pasture. Another study measuring cow motivation for pasture access used three different distances, with the two shorter distances being similar to the Medium and Long distance in the current study [28]. That study found that cows spent less time on and less time lying on pasture compared to the open lying areas in the current study. As that study only recorded lying times during the day, it is not surprising that, at similar walking distances, the lying times were lower than those recorded in the current study for the open lying areas. Additionally, that previous study found an effect of rain on cow motivation to go out to pasture [28], which may explain why cows spent less time at pasture at a similar distance compared to the current study, which took place indoors. It is possible that cow motivation to lie down on an open lying area in the form of pasture or on an open lying area indoors is similar, however without knowing whether a ceiling effect exists for either lying option, we cannot be certain which option for an open lying area cows find the most attractive.

Cows in the current study had longer lying bouts on the open lying areas compared to the cubicles, which can account for longer overall lying times on the open lying surfaces compared to the cubicles [42]. However, longer lying bouts are generally reported on harder lying surfaces [43], as cows experience discomfort in the process of lying down and getting up and therefore are reluctant to get up once lying down [44,45]. In the current study, the lying surfaces of the open lying areas offered were at least as soft as the cubicles. When straw is added to a rubber mattresses, lying bout durations are increased with each kg added and improves cow comfort as the surface is more compressible [46]. In a study investigating the lying behaviour of dairy cows in different cubicle sizes, cows had longer lying bouts in larger cubicles [13]. Additionally, a study showed that cows have longer lying bouts when offered more space per cow on open rubber matting [47]. Therefore, the increase in lying bout duration in the current study on the open lying surfaces compared to the cubicles could be interpreted as a response to access to increased lying space, rather than in indicator of a lack of surface comfort on the open lying areas.

Cows spent longer lying down on the open lying surfaces at night compared to in the day-time in the current study. Cows exhibit a clear diurnal lying pattern, with cows lying down primarily at night [42,48], which has been observed at pasture [18] and in cubicles [49]. Motivation for the open lying areas in the current study was higher at night compared to the day, which is similar to diurnal patterns shown in cow motivation for pasture access [26,28,29]. However, time spent lying down at night on the open lying areas did decrease with increasing distance. This was not found to be the case in the previous pasture motivation studies mentioned, which found lying at pasture at night did not change with the increased cost of access. This might partly be accounted for by how day-time and night-time were calculated in the current study, using the time of sunrise and sunset, to adjust for the seasons. Previous pasture motivation studies have used a fixed time point throughout the study to differentiate between night-time and day-time, am and pm milkings [26,28,29], an option not available with a milking robot. Additionally, previous pasture motivation studies took place during a relatively seasonally consistent period (27: May–July 2010; 28: July–September 2011). In the current study, time spent lying down on the open lying areas at night increased during the transition from Autumn to Winter (between experimental periods 1–4) before decreasing as the seasons moved from Spring into Summer (experimental periods 5 and 6), possibly caused by the overall change in night length. Had a fixed point been used to differentiate day-time and night-time, this observational effect of experimental period may not have been seen. However, without consistent parlour milking times and a large difference between daylight hours throughout the study, the authors felt that this was the most sensible method to define night and day.

Activities such as eating, drinking and sleeping are said to be ‘resilient’ activities which animals tend to show inelastic demand to perform, meaning that an animal will continue to work to perform such activities despite an increasing cost [12,50]. Lying behaviour in cows has been shown to have an inelastic demand, with heifers and cows working to lie down for between 12–13 h/d [11,51], although this can fluctuate within lactation [52]. Studies measuring cow motivation for lying down found that as the workload to access an area to lie down increased, time spent on other behaviours decreased [6,11]. Although the current study is not an example of a true demand type study (cows had unlimited access the open lying area after paying the price for access), a similar result was found. Cows in the current study spent a smaller proportion of their time in the open lying areas performing activities other than lying down at the Long distance, compared to the Short and Medium distances, where time spent on these activities was higher. This highlights that although lying time decreased on the open lying areas at the Long distance, lying down became a more important behaviour to perform after paying the higher price for access.

## Conclusions

Cows in the current study were motivated to access and lie down in the open lying areas compared to the cubicles, with cows having a higher motivation, in terms of time spent lying, for an open deep-bedded straw area than an open cow mattress. Surface type had a smaller effect on motivation than walking distance, demonstrating the value cows place on an open lying area regardless of the surface type in the current study. Given cows value access to open lying areas, the provision of such areas has the potential to improve cow welfare in commercial housing systems in the future.

## Supporting information

S1 Table Animal details. Mean (± SEM) and range of lactation number, days in milk, milk yield (kg/d), body condition score and lameness score for all cows in the study. * This study included 6 primiparous and 24 multiparous cows.
(PDF)

S2 Table Experimental periods. Start and end date for each experimental period (1–6) within the study.
(PDF)

S3 Table Results summary. Summary, averaged for all cows, of time spent lying (h/d), lying bout frequency per day, lying bout duration (hr), time spent lying during the day (h/d), and time spent lying during the night (h/d) on MAT, ST, Open Lying Surfaces and Cubicles at the Short, Medium and Long distances and time spent not lying (h/d) and raceway completion frequency per day for ST and MAT at the Short, Medium and Long distance (± SEM).
(PDF)

S1 File Training protocol. A detail description of the training protocol.
(PDF)

S1 Dataset Master dataset. A collated dataset for the behavior of each cow during each choice period (3 d) and a table of content (ToC) for the dataset.
(XLSX)
