# Supplementary material for: Dairy cows value an open area for lying down
Source: PLoS One. 2022 May 27;17(5):e0268238. doi: 10.1371/journal.pone.0268238 (PMC9140234; doi:10.1371/journal.pone.0268238)
Supplement: S1 Table — Mean (± SEM) and range of lactation number, days in milk, milk yield (kg/d), body condition score and lameness score for all cows in the study. * This study included 6 primiparous and 24 multiparous cows. (PDF) [file pone.0268238.s001.pdf]

	**Mean (± SEM)**	**Min**	**Max**
Lactation Number *	2.8 ± 0.2	1	6
Days In Milk	260 ± 15.5	130	466
Milk Yeild (kg/d)	24.13 ± 1.84	1.78	42.26
Body Condition Score	3 ± 0.03	2.75	3.5
Lameness Score	1.62 ± 0.04	1.5	2
