# Supplementary material for: Dairy cows value an open area for lying down
Source: PLoS One. 2022 May 27;17(5):e0268238. doi: 10.1371/journal.pone.0268238 (PMC9140234; doi:10.1371/journal.pone.0268238)
Supplement: S2 Table — Start and end date for each experimental period (1–6) within the study. (PDF) [file pone.0268238.s002.pdf]

Experimental Period No.	Start Date	End Date
1	August 31st, 2019	October 1st, 2019
2	October 5th, 2019	November 5th, 2019
3	November 9th, 2019	December 10th, 2019
4	January 11th, 2020	February 11th, 2020
5	February 15th, 2020	March 17th, 2020
6	June 20th, 2020	July 21st, 2020
