# Supplementary material for: Dairy cows value an open area for lying down
Source: PLoS One. 2022 May 27;17(5):e0268238. doi: 10.1371/journal.pone.0268238 (PMC9140234; doi:10.1371/journal.pone.0268238)
Supplement: S3 Table — Summary, averaged for all cows, of time spent lying (h/d), lying bout frequency per day, lying bout duration (hr), time spent lying during the day (h/d), and time spent lying during the night (h/d) on MAT, ST, Open Lying Surfaces and Cubicles at the Short, Medium and Long distances and time spent not lying (h/d) and raceway completion frequency per day for ST and MAT at the Short, Medium and Long distance (± SEM). (PDF) [file pone.0268238.s003.pdf]

	Short	Medium	Long
**MAT**			
Lying (h/d)	10.3 ± 0.9	9.0 ± 1.0	6.7 ± 1.0
Lying Bout Frequency (per day)	7.2 ± 0.7	6.7 ± 0.7	4.6 ± 0.8
Lying Bout Duration (h)	1.4 ± 0.1	1.3 ± 0.1	1.2 ± 0.1
Lying Day (h/d)	3.4 ± 0.4	3.0 ± 0.4	2.3 ± 0.4
Lying Night (h/d)	6.7 ± 0.6	6.0 ± 0.7	4.5 ± 0.7
Not Lying (h/d)	1.5 ± 0.3	1.1 ± 0.2	0.7 ± 0.1
Raceway Completion Frequency (per day)	3.6 ± 0.4	2.7 ± 0.3	1.8 ± 0.3
**ST**			
Lying	12.4 ± 0.6	11.3 ± 0.9	8.2 ± 1.0
Lying Bout Frequency	8.5 ± 0.5	7.4 ± 0.6	5.2 ± 0.7
Lying Bout Duration	1.5 ± 0.1	1.5 ± 0.1	1.4 ± 0.1
Lying Day	4.3 ± 0.4	3.6 ± 0.4	2.8 ± 0.4
Lying Night	8.2 ± 0.4	7.6 ± 0.6	5.5 ± 0.7
Not Lying	1.3 ± 0.1	0.9 ± 0.1	0.7 ± 0.1
Raceway Completion Frequency (per day)	4.3 ± 0.2	3.3 ± 0.3	2.1 ± 0.3
**Open Lying Surfaces**			
Lying	11.4 ± 0.6	10.1 ± 0.7	7.5 ± 0.7
Lying Bout Frequency	7.9 ± 0.6	7.0 ± 0.6	4.9 ± 0.7
Lying Bout Duration	1.4 ± 0.1	1.4 ± 0.1	1.3 ± 0.1
Lying Day	3.8 ± 0.3	3.3 ± 0.3	2.5 ± 0.5
Lying Night	7.5 ± 0.4	6.8 ± 0.5	5.0 ± 0.3
**Cubicles**			
Lying	2.3 ± 0.5	3.1 ± 0.5	4.8 ± 0.6
Lying Bout Frequency	2.1 ± 0.4	2.9 ± 0.5	4.7 ± 0.6
Lying Bout Duration	0.6 ± 0.1	0.7 ± 0.1	0.9 ± 0.1
Lying Day	1.1 ± 0.2	1.2 ± 0.2	1.9 ± 0.3
Lying Night	1.3 ± 0.3	1.9 ± 0.2	2.9 ± 0.3
