# Supplementary material for: Dairy cows value an open area for lying down
Source: PLoS One. 2022 May 27;17(5):e0268238. doi: 10.1371/journal.pone.0268238 (PMC9140234; doi:10.1371/journal.pone.0268238)
Supplement: S1 File — A detail description of the training protocol. (PDF) [file pone.0268238.s004.pdf]

- ‘Day 1’ of this training protocol occurred on day 4 as per Fig 2, ‘Day 2’ as per day 5, and ‘Day 3’ on every subsequent training day (day 6, 10, 14, 18, 19, 20, 24 and 28).
- At any point during training days, if all cows entered the raceway, lay down in the open space and exited of their own accord, that group of cows were considered trained for that training period and were given free access to the experimental area for the remainder of that training period.

Day 1

- 9am – 2pm: Cows had access to the ‘Experimental Area’ with the raceway at the Short distance and left to explore for themselves.
  - The first one-way gate to enter the open lying areas was tied open to encourage the cows through, as they had not encountered this type pf one-way gate before and needed to learn to push it.
  - The second one-way to exit to the ‘Cubicle Area’ was left closed i.e. cows needed to push it to get through it.
  - Cows that did not learn to push the one-way gate to exit to the ‘Cubicle Area’ during this exploration time were still able to return to the ‘Cubicle Area’ via the first, tied open one-way gate i.e. they were not confined to the ‘Experimental Area’.
- 2pm – 3pm: Any cows in the ‘Cubicle Area’ were encouraged through the raceway and the first one-way gate (tied open) into the open lying area. The first one-way gate was then closed and the cows left to explore the open lying area for 5 minutes in case some cows had not explored the area themselves before this. The cows were then encouraged to push through the second one-way gate, back into the ‘Cubicle Area’.

    - 3pm – 5pm: The first one-way gate was tied back open and cows had free access between the ‘Cubicle Area’ and the ‘Experimental Area’.
- 5pm -6pm: Any cows left in the open lying area were encouraged to push through the second one-way gate, back into the ‘Cubicle Area’. All cows were then encouraged through the raceway and the first one-way gate (tied open) into the open lying area.
    - Cows had free access over night between the ‘Cubicle Area’ and the ‘Experimental Area’.

Day 2

- The first one-way gate to enter the open lying areas was loosened and tied to half way between closed and open. This was closed enough that it functioned as a one-way gate, but open enough to encourage cows to push through.
- Any cows in the ‘Experimental Area’ were encouraged to push through the second one-way gate, back into the ‘Cubicle Area’ for the duration of cleaning.
- 9am – 10am: All cows were encouraged through the raceway and the first one-way gate (tied half way between closed and open) into the open lying area.
    - 10am – 12pm: Cows had free access between the ‘Cubicle Area’ and the ‘Experimental Area’.
- 12pm – 1pm: Any cows left in the open lying area were encouraged to push through the second one-way gate, back into the ‘Cubicle Area’. All cows were then encouraged through the raceway and the first one-way gate (tied half way between closed and open) into the open lying area.
    - 1pm – 3pm: Cows had free access between the ‘Cubicle Area’ and the ‘Experimental Area’.
- The first one-way gate was untied.

- 3pm -4pm: Any cows left in the open lying area were encouraged to push through the second one-way gate, back into the ‘Cubicle Area’. All cows were then encouraged through the raceway and the first one-way gate (untied) into the open lying area.
    - Cows had free access over night between the ‘Cubicle Area’ and the ‘Experimental Area’.

Day 3

- Any cows in the ‘Experimental Area’ were encouraged to push through the second one-way gate, back into the ‘Cubicle Area’ for the duration of cleaning.
- 9am – 10am: All cows were encouraged through the raceway and the first one-way gate into the open lying area.
    - 10am – 12pm: Cows had free access between the ‘Cubicle Area’ and the ‘Experimental Area’.
- 12pm – 1pm: Any cows left in the open lying area were encouraged to push through the second one-way gate, back into the ‘Cubicle Area’. All cows were then encouraged through the raceway and the first one-way gate into the open lying area.
    - 1pm – 3pm: Cows had free access between the ‘Cubicle Area’ and the ‘Experimental Area’.
- 3pm -4pm: Any cows left in the open lying area were encouraged to push through the second one-way gate, back into the ‘Cubicle Area’. All cows were then encouraged through the raceway and the first one-way gate into the open lying area.
    - Cows had free access over night between the ‘Cubicle Area’ and the ‘Experimental Area’.
